# Gene expression reveals two distinct groups of anal carcinomas with clinical implications

**DOI:** 10.1038/sj.bjc.6604285

**Published:** 2008-03-18

**Authors:** O Bruland, Ø Fluge, H Immervoll, L Balteskard, M P Myklebust, A Skarstein, O Dahl

**Affiliations:** 1 Center of Medical Genetics and Molecular Medicine, Haukeland University Hospital, Bergen 5021, Norway; 2 Department of Oncology, Haukeland University Hospital, Bergen 5021, Norway; 3 Department of Pathology, The Gade Institute, Haukeland University Hospital, Bergen 5021, Norway; 4 Section for Pathology, University of Bergen, Bergen 5021, Norway; 5 Section of Oncology, University Hospital of Northern Norway, Tromsø 9038, Norway; 6 Department of Surgery, Haukeland University Hospital, Bergen 5021, Norway; 7 Department of Surgical Sciences, University of Bergen, Bergen 5021, Norway; 8 Section of Oncology, Institute of Medicine, University of Bergen, Bergen 5021, Norway

**Keywords:** anal cancer, human papillomavirus, microarray, gene expression profiling, MCM7, CDKN2A (p16)

## Abstract

Human papillomavirus (HPV) is a major aetiological agent in anal carcinomas. We here present a study of global gene expression using microarray hybridisation in a collection of anal carcinoma biopsies. Quantitative PCR was used to verify expression of selected genes. All biopsies contained integrated DNA of human papillomavirus subtype 16 (HPV16) and expressed HPV16 E7 mRNA. No other subspecies of HPV were detected in these 13 biopsies as assessed by PCR amplification and DNA sequencing. Unsupervised cluster analysis, based on global mRNA expression, divided the tumour biopsies into two distinct groups. Cluster analysis based on a number of high-risk HPV and/or E2F-regulated genes reproduced this biopsy grouping, suggesting that integrated HPV16 substantially influenced global gene expression in approximately half the biopsies studied. The levels of HPV16 E7 mRNA were significantly different between the two groups, but with considerable overlap. Thus, influence on global gene expression could not be absolutely ascribed to the expression level of HPV16. To investigate whether this distinction in gene expression had prognostic impact, we studied protein expression in an independent cohort of 55 anal carcinomas not included in the microarray study of two differentially expressed candidate genes, minichromosome maintenance complex component 7 (MCM7) and cyclin-dependent kinase inhibitor 2A (CDKN2A or p16). HPV status was assessed by *in situ* hybridisation. There was a significant association between *in situ* staining for HPV E7 mRNA and immunostaining for CDKN2A (p16) and MCM7 protein. CDKN2A (p16) mRNA was found significantly differentially expressed between the two tumour groups. However, cluster analysis on genes directly regulated by CDKN2A (p16) could not reproduce this split of biopsies into two groups, suggesting that the transcriptional regulatory activity of CDKN2A in these biopsies is inhibited. Furthermore, protein expression of CDKN2A (p16) could not be associated with survival. MCM7 is directly regulated by E2F and induced by HPV, and its mRNA was found differentially expressed between the two tumour groups. High level of MCM7 protein was found to be associated with both improved relapse-free survival (RFS, *P*=0.02) and cancer-specific survival (CSS, *P*=0.03) in anal cancer patients treated with radiation with or without additional chemotherapy.

It is widely accepted that the aetiological agent of the large majority of anal squamous cell carcinomas (SCCs) is the human papillomavirus (HPV) (zur Hausen, 1989; Frisch *et al*, 1997; Goedert *et al*, 1998; Daling *et al*, 2004). Other mechanisms, such as immunosuppression, dramatically increase the risk for developing anal cancer (Penn, 1986; Arends *et al*, 1997). The HPV family comprises numerous epitheliotropic subtypes affecting the skin, the oropharyngeal or the anogenital mucosa. Some mucosal HPV subtypes, mainly human papillomavirus subtype 16 (HPV16) and human papillomavirus subtype 18 (HPV18), are considered high risk for neoplastic development (Munoz *et al*, 2003) and express the viral oncogenes E6 and E7 with transforming properties (zur Hausen, 2000). The transforming potential of E6 and E7 are, at least in part, based on their inactivation of both p53 by the E6 protein (Werness *et al*, 1990) and the retinoblastoma (RB1) protein by E7 (Dyson *et al*, 1989) leading to genomic instability, inadequate G1/S checkpoint control and antiapoptosis. The oncogenic potential of high-risk HPV is probably activated by the integration of viral genome into the host cell genome. When this integration leads to disruption of the HPV E2 gene, it results in elevated expression of E6 and E7 and, consequently, enhanced degradation of the host cell tumour repressor proteins p53 and RB1 (Alani and Munger, 1998; Psyrri *et al*, 2004). In addition, cells that express E6/E7 from integrated HPV sequences have a selective growth advantage over cells with episomal HPV genomes (Jeon *et al*, 1995). The concept that loss of E2 repressor function may be critical for malignant progression is supported by experiments showing that re-expression of E2 in cervical cancer cell lines causes growth suppression (Thierry and Yaniv, 1987). Other factors, including sequence variants of HPV subtypes, seem to be important for the oncogenic potential of the virus (Fujinaga *et al*, 1994; Ellis *et al*, 1995; Hecht *et al*, 1995).

The retinoblastoma protein (pRB) pathway plays a central role in regulating the progression through the G1 phase of the mammalian cell cycle (Sherr, 1996). The core members of this pathway include, in addition to pRB (and its family members p107 and p130), the D-type cyclins that, in association with CDK4 and CDK6, promote proliferation of the cell cycle and the INK4 family of cyclin-dependent kinase inhibitors (including cyclin-dependent kinase inhibitor 2A (CDKN2A) or p16) that specifically binds and inhibits the activity of CDK4 and CDK6. Also, the most studied and the best-understood targets for pRB are members of the E2F transcription factor family (Dyson, 1998; Muller *et al*, 2001). The transcription factor E2F is a key transcriptional regulator belonging to a family of transcription factors responsible for transcriptional control of genes encoding proteins involved in several biological processes (Cobrinik, 2005). This includes DNA replication, enzymes involved in deoxynucleotide biosynthesis, proteins that assemble to form functional origin of replication complexes, and kinases involved in the activation and initiation of cell-cycle progression (Ishida *et al*, 2001) as well as transcription of negative regulators of cell-cycle progression such as CDKN2C (p18) (Blais *et al*, 2002). The high frequency of alterations that have been identified in the pRB pathway in human cancer, a pathway central for the regulation of cell proliferation, suggest that the deregulation of the pRB pathway is an obligatory event in human cancer (Hanahan and Weinberg, 2000).

Given the many HPV subtypes, and the transforming function being dependent on sequence variants and integration events, establishing the oncogenic potential in a given HPV-infected cancer is a complex task. Rather than identifying the exact viral species or variant, we propose that it will be advantageous to investigate the effects of the integrated virus on global gene expression, thus possibly establishing potential markers that can be associated with disease outcome. Although HPV is strongly associated with anal cancer (Daling *et al*, 2004), it is not known how the presence of high-risk HPV affects global gene expression in anal cancer *in vivo*. To elucidate potential prognostic or predictive markers for HPV-associated anal cancer, we analysed the mRNA expression profile in 13 HPV16-positive anal cancer biopsies and 4 HPV16-negative normal anal mucosa samples. Furthermore, we tested the associations between protein expression of two isolated target genes, MCM7 and CDKN2A (p16) with HPV E7 mRNA expression and with the clinical outcome in 55 anal cancer patients.

## MATERIALS AND METHODS

### Patient and tissue samples

#### Microarray expression analysis

The biopsies used for gene expression analyses were taken from 13 patients with anal carcinoma, prior to treatment, and are summarised in Table 1. One sample was obtained at the University Hospital of Tromsø (Tromsø, Norway). The rest of the samples were obtained at Haukeland University Hospital (Bergen, Norway). Eleven of these patients were women. The mean age was 66.3 years (range: 45–88 years). The histology was SCC in all, except for one case with Paget's disease (ca-29), and one had a basaloid type of SCC (ca-36). The TNM stage for each patient is given in Table 1. All patients except ca-20 and ca-47 were treated with radiation therapy combined with concomitant chemotherapy using mitomycin C (10 mg m^−2^) and 5-fluorouracil (1000 mg m^−2^, days 1–4). Patient ca-20 was a 63-year-old woman presenting with T4N2 disease, and patient ca-47 was a 54-year-old man with T3N1 disease and a possible retroperitoneal lymph node metastasis (M1). These two had first two courses of chemotherapy with 5-fluorouracil and cisplatin (80 mg m^−2^) with 3 weeks interval, and then they were given concomitant chemotherapy with cisplatin (60 mg m^−2^) and 5-fluorouracil during the first week of radiation therapy. Primary surgery was not performed in the 13 patients. The mean follow-up was 40 months (range: 25–58 months for patients alive).

Three patients had a relapse (as indicated in Table 1) and died from anal carcinoma. Patient ca-41 had a distant relapse 52 months from diagnosis and died 3 months later. Patient ca-24 had a local relapse at 11 months and died 1 month later, while patient ca-47 (with possible M1 disease initially) died from distant metastases 31 months after diagnosis. Among the 10 nonrelapsing patients, two have experienced breast cancer during follow-up (patients ca-22 and ca-39).

Fresh tissue samples were taken with a conchotome, directly snap-frozen in liquid nitrogen and stored at −80°C. All biopsy specimens were examined by a histopathologist and classified according to WHO criteria (Hermanek, 1992). Clinical data were collected from the hospital files. Cytological smears obtained from the biopsies immediately prior to tissue lysis and RNA extraction were evaluated for the presence of tumour cells.

#### Tissue microarray

Independent samples of formalin-fixed and paraffin-embedded (FFPE) tumour tissues from the time of diagnosis, prior to therapy, from 62 patients with anal carcinoma were selected from a previous patient cohort. A tissue microarray (TMA) was performed as described below. Seven of them were treated with surgery alone. The 55 patients included in the survival analyses were treated with radiation alone or combined with concomitant chemotherapy (5-fluorouracil (1000 mg m^−2^ days 1–4) and mitomycin C (10 mg m^−2^) (*n*=30); UKCCCR ACTWP, 1996; Bartelink *et al*, 1997). Among these 55 patients, the median radiation dose was 50 Gy (range: 28–60 Gy). Of 46 patients treated with concomitant chemotherapy, 28 received one course and 18 patients received two courses of chemotherapy.

Mean follow-up time was 7.2 years.

### RNA extraction

Total RNA was extracted using the Qiagen RNeasy minikit (QIAGEN GmbH, Hilden, Germany), as recommended by the manufacturer. The first flow-through fraction was collected for subsequent DNA extraction. Three micrograms of total RNA was subjected to DNase I treatment to remove any contaminating genomic DNA, using TURBO DNase™ (Ambion, cat. no. 2238). The RNA quality and quantity were evaluated by Agilent Bioanalyzer and OD260-280, respectively.

### DNA extraction

Genomic DNA was extracted from the first flow-through following the RNA purification protocol. Briefly, 350 *μ*l flow-through were mixed with 1 *μ*l linear acrylamide (Ambion, cat. no. 9520) and 500 *μ*l 100% EtOH, vortexed and centrifuged at 13 000 **g** in a tabletop centrifuge. The precipitate was air-dried, and subsequently purified by Qiagen DNA minikit (cat. no. 13323).

### Detection of genomic human papillomavirus DNA

Quantification of HPV16 and 18 DNA was done as described (Lamarcq *et al*, 2002). All samples were run in triplicate. Estimation of the relative copy number of HPV16 or HPV18 DNA was performed by the comparative CT method relative to the peripheral myelin protein 22 gene (pmp22). In addition, subspecies of HPVs were assessed by PCR amplification with degenerate PCR primers (MY09 and MY11) (Manos *et al*, 1989) followed by DNA sequencing (data not shown).

### Microarray

Gene expression analysis was performed on the ABI 1700 Expression array system (ABI, Foster City, CA, USA) with the Applied Biosystems Chemiluminescent RT-IVT Labelling Kit (cat. no. PN 4339629) and Human Genome microarray (ABI, cat. no. 4337467) using 2 *μ*g high-quality total RNA. Fifteen micrograms of mRNAs were hybridised to the ABI microarray. The Applied Biosystems Human Genome Survey Microarray v. 1.0 provides 31 700 probes for interrogation of 27 868 genes. Each probe is a synthetic oligodeoxynucleotide of 60 nucleotides (nt). The oligonucleotide probes are modified at the 3′ end by the addition of a C-6 spacer and are covalently attached to the support via a terminal amino group. In almost all cases, probes are placed within 1500 nt of the 3′ end, where labelling is more robust. The Applied Biosystems 1700 Chemiluminescent Microarray Analyzer is a component of the integrated Applied Biosystems Expression Array System, which uses the power and sensitivity of chemiluminescence to identify and measure gene expression levels.

All procedures were performed according to the manufacturer's recommendations.

### Analysis of microarray data

J-Express Pro v. 2.7 (Dysvik and Jonassen, 2001) was used for all filtering, clustering and statistical analysis of the microarray experiments. The compiled set consisted of 22 385 individual probes. Euclidean hierarchical clustering with average linkage (weighted pair group method with arithmetic mean, WPGMA) was performed on this data set for the total unsupervised clustering. This unsupervised clustering divided the tumour biopsies into two distinct clusters, group 1 (Gr1) and group 2 (Gr2). Biopsies belonging to Gr1 were labelled in blue and in Gr2 labelled in red and are consistently illustrated so throughout this paper. Additional filtering left 8449 spots remaining. All further analysis was done on this set. To isolate the genes accounting for the observed clustering, we grouped the biopsies according to the result of the unsupervised cluster analysis and performed supervised *T*-test and significance of microarray (SAM) (Tusher *et al*, 2001) analysis of the two groups (Gr1 and Gr2), in both cases returning 500 probes with the lowest *P*-value. The resulting 500 probes from the *T*-test analysis were sorted according to the biological process classification by the Human protein reference database (HPRD) (http://www.hprd.org/). Of the 500 probes, 344 returned a unique HPRD reference, the rest being either duplicate probes or hypothetical genes that had no reference in this particular database. The HPRD classification divided the 344 probes into eight distinct biological processes. Euclidean hierarchical clustering with average linkage was performed for each of the eight subgroups.

### Quantitative real-time PCR analysis

Sequence-specific primers and TaqMan probes for HPV16 and HPV18 E7 mRNA and DNA were used as described by others (Lamarcq *et al*, 2002). For verification of microarray results with real-time MGB Quantitative PCR, we used the following Applied Biosystems AssayOnDemand probes: CHAF1B, chromatin assembly factor 1, subunit B (p60) (Assay ID Hs00601414_m1); MCM7, minichromosome maintenance complex component 7 (Hs00428518_m1); MSH6, mutS homolog 6 (*Escherichia coli*) (Hs00264721_m1); PSIP1, PC4 and SFRS1 interacting protein 1 (Hs00253515_m1); KIF2C, kinesin family member 2C (Hs00199232_m1); LMNB1, lamin B1 (Hs00194369_m1); LMNB2, lamin B2 (Hs00383326_m1); MAPKAPK3, MAP kinase-activated protein kinase 3 (Hs00177957_m1); GAS7, growth arrest-specific 7 (Hs00245902_m1); CCNB1, cyclin B1 (Hs00259126_m1); CCNA2, cyclin A2 (Hs00153138_m1); CCNB2, cyclin B2 (Hs00270424_m1); CDKN2C, cyclin-dependent kinase inhibitor 2C (p18) (Hs00176227_m1); MDK, midkine (neurite growth-promoting factor 2) (Hs00171064_m1); BNIPL, BCL2/adenovirus E1B 19 kD interacting protein like (Hs00370514_m1); and Bax, BCL2-associated X protein (Hs00180269_m1). Statistical comparisons were performed using the Mann–Whitney rank sum test with the GraphPad Pris™ v. 4.0 software (GraphPad Software Inc., San Diego, CA, USA). *P*-values were two-sided and considered significant when <0.05.

### Tissue microarray

Sixty-two formalin-fixed, paraffin-embedded tumour samples of representative tumour regions from individual patients were used for the preparation of a tumour TMA block. The TMA block was constructed using a Manual Tissue Arrayer 1 (Beecher Instruments, Silver Spring, MD, USA) with a 1.0-mm diameter core biopsy needle. A pathologist reviewed the H&E-stained slides to control for tissue quality and confirm the diagnosis. Following the construction of the array block, 5 *μ*m sections were cut with a microtome. Each slide used for immunohistochemistry (IHC) was de-paraffinised and rehydrated following standard protocols.

#### Immunohistochemistry

For the detection of MCM7 protein, target retrieval was performed in 0.05% citraconic anhydride solution, pH 7.4 (Namimatsu *et al*, 2005) at 121°C for 15 min. Immunohistochemistry was performed using an MCM7-specific monoclonal antibody (47DC141, cat. no. ab2360; Abcam plc, Cambridge, UK) diluted 1 : 200 in Antibody Diluent solution (cat. no. S0809, Dako Denmark, Glostrup, Denmark). For the detection system, the Polymer-HRP IHC Ready-to-Use Detection System/DAB kit (no. QD400, Biogenex, San Ramon, CA, USA) was used according to the manufacturer's recommendations. Finally, colour was developed using 3,3′-diaminobenzidine (DAB; Dako) and counterstained with haematoxylin (Dako; ChemMate™ Hematoxylin no. S-2020).

For the detection of CDKN2A (p16) protein, target retrieval was performed in 10 mM citrate-buffer pH 6 in a microwave oven. Immunohistochemistry was performed using a CDKN2A (p16)-specific antibody (BD Biosciences, Sparks, MD, USA, P/N 554070) diluted 1 : 200 in Antigen Diluent Solution, using a Dako Autostainer and the EnVision DAB detection system (Dako, P/N K5007).

The sections were dehydrated and mounted using Eukitt® quick-hardening mounting medium (Fluka, Industriestrasse, Buchs, Switzerland, cat. no. 03989).

Immunohistochemical analysis of p16 and MCM7 expression was scored independently by two of the authors. The staining intensity of MCM7 was categorised as negative (0), weak (1), moderate (2) or strong (3). An index was made from the product of staining intensity (0–3) and from the percentage of positive tumour cells (0–100). This index then was categorised as low (index <140, *n*=27) or high (index ⩾140, *n*=28). The cutoff value of 140 was the median value of the complete data set, dividing the tumour biopsies essentially into two equal numbered groups. The impact of the protein-staining index on clinical outcome then was assessed on the 55 patients treated with radiation with or without additional chemotherapy (that is the 7 patients treated with surgery alone were left out). The Kaplan–Meier method (Bland and Altman, 1998) was used to estimate relapse-free survival (RFS) and cancer-specific survival (CSS), and the log-rank method was used to compare survival between groups.

### *In situ* hybridisation

The HPV *in situ* hybridisation (ISH) was performed using reagents and INFORM® HPV III Family 16 DNA Probe (B) (P/N 800-4295) provided by Ventana Medical Systems Inc. (Tucson, AZ, USA). Developed to stain FFPE tissue sections, this probe is designed to detect HPV genotypes 16, 18, 31, 33, 35, 39, 45, 51, 52, 56, 58 and 66. This probe was used in conjunction with a Discovery automated slide stainer, the RiboMap kit (P/N 760-102) and BlueMap-detection kit (P/N 760-120), using a streptavidin alkaline phosphatase conjugate that gives a blue colour in the chromogenic reaction with NBP (nitroblue terazolium) and BCIP (5-bromo-4-chloro-indolyl-phosphate). The 5-*μ*m-thick sections were de-paraffinised and rehydrated on the stainer. DNA unmasking was performed with RiboCC buffer (P/N 760-107) and protease 3 (P/N 760-2020) for 20 min. A mix of 200 *μ*l HPV III probe, 75 *μ*l RiboHybe (P/N 760-104) and 25 *μ*l 2 × SSC was applied on each slide for hybridisation. The probe and target were denatured at 95°C for 8 min and incubated for 2 h at 52°C for hybridisation. Stringent wash were performed in 2 × SSC at 72°C. A 20-min incubation with a rabbit anti-DNP antibody (P/N 780-4335) was included prior to the 20-min incubation with the Universal Secondary Antibody (P/N 760-4205). The slides were counterstained with ISH Red Counterstain (P/N 780-2186), dehydrated through graded alcohols and xylene, and mounted. HPV-positive cervix tumour was used as a positive control. A negative control, omitting the probe, was included.

For HPV-ISH, nucleolar E7 mRNA was scored as negative, weak, moderate, strong or intense by two independent investigators. For analysis of association between MCM7 protein staining and E7 mRNA, E7 mRNA was recoded as negative or positive, and analysed by *χ*^2^ statistics including all 62 patients.

The Regional Ethics Committee Region West approved this study.

## RESULTS

### Patient characteristics

Histopathological evaluation of the biopsies used for RNA extraction is described in Table 1. Biopsies on the tissue array included 62 patients with histologically confirmed primary anal SCC.

### Identification of HPV virus and evaluation of HPV16 viral load

**Figure 1:**
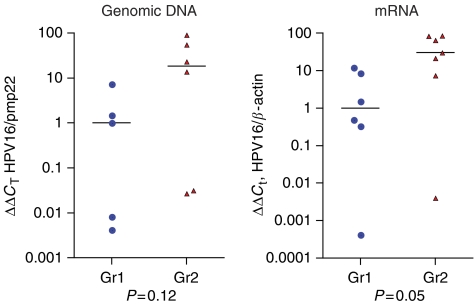
Taqman quantitative PCR for Gr1 and Gr2 anal cancer biopsies of the relative amounts of HPV16 E7 DNA relative to the single-copy gene pmp22 (*P*=0.12) and mRNA relative to *β*-actin mRNA (*P*=0.05).

All cancer biopsies included in the microarray study tested positive for HPV16 mRNA by quantitative real-time PCR analysis (Figure 1), while the four normal mucosa biopsies tested negative. No other subspecies of HPV virus were detected in this selection of biopsies when performing PCR amplification with the degenerate primers followed by DNA sequencing. The relative amount of HPV16 E7 mRNA was higher in Gr2 compared with Gr1 (*P*=0.05). In two biopsies, ca-29 (Gr1) and ca-24 (Gr2), only small amounts of HPV E7 mRNA were detected. For two of the biopsies (ca-3 in Gr1 and ca-11 in Gr2), we were unable to isolate genomic DNA. For the rest of the biopsies, the relative amount of viral DNA was estimated by comparison to the single-copy gene, pmp22. The estimated copy number of HPV16 genomes compared with host cell genome varied from approximately six copies per cell to one copy per 20 cells (HPV16 genome: host cell genome) (Figure 1).

### Microarray analysis

**Figure 2:**
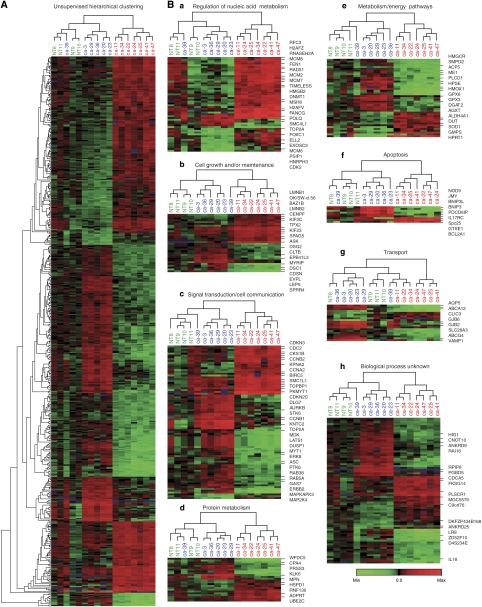
(**A**) Hierarchical clustering of all 337 genes selected by performing a *T*-test between the two tumour groups. (**B**) Distribution of 337 genes into eight different biological processes, as defined by the Human Protein Reference Database (HPRD) (**Ba**–**Bh**). A heat map of the corresponding hierarchical clustering illustrates the relation between all biopsies in each individual subgroup. The locations in each individual cluster of a number of selected genes are indicated. The colour scale indicates the range of relative expression levels, red representing upregulated and green representing downregulated expression, both as compared to the reference. See online version for colour figure.

**Figure 3:**
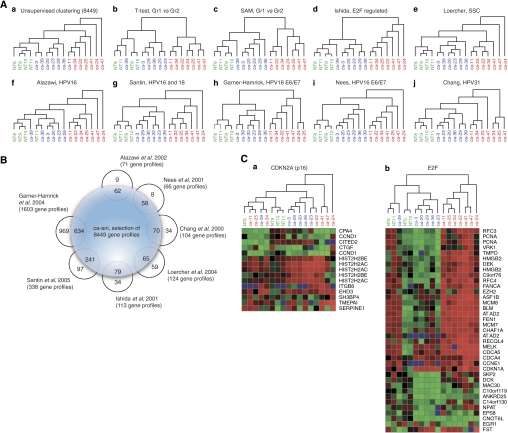
(**A**) Hierarchical clustering of the unsupervised analysis on (**Aa**) the selection of 8449 probes, (**Ab**) the 337 individual genes defined by the *T*-test, (**Ac**) the 363 individual genes defined by the SAM analysis together with collections of genes described by others relevant to our study and included within the group of 8449 probes, (**Ad**) HPV16-induced gene expression (Alazawi *et al*, 2002), (**Ae**) HPV16 E6/E7-induced gene expression (Nees *et al*, 2001), (**Af**) HPV31-induced gene expression (Chang and Laimins, 2000), (**Ag**) Gene expression in SSC (Loercher *et al*, 2004), (**Ah**) E2F-regulated genes (Ishida *et al*, 2001), (**Ai**) HPV16- and HPV18-induced gene expression (Santin *et al*, 2005) and, finally, (**Aj**) HPV18 E6/E7-induced gene expression (Garner-Hamrick *et al*, 2004). (**B**) The total number of genes within each report cited. The numbers listed in parenthesis following the author are the total number with matching probes located on the ABI microarray. The number inside the large circle is the number of genes with matching probes in the 8449 selection. The number outside the big circle corresponds to genes with matching probes on the ABI chip but not included in the 8449 selection. (**C**) Hierarchical cluster analysis based on (**Ca**) CDKN2A (p16) transcriptionally regulated genes and (**Cb**) E2F-regulated genes, as reported by Vernell *et al*, 2003.

Unsupervised hierarchical clustering on 22 385 genes readily distinguished tumours from the normal anal mucosa, and further identified two main subclasses of anal carcinomas based on distinct patterns of gene expression (Figure 3Aa). Further filtering, eliminating genes not differentially regulated between the samples, reduced the total number of probes to 8449. When performing a SAM analysis (Tusher *et al*, 2001) on this set, comparing the normal tissue group with the tumour group, 1296 probes showed a difference in expression level of more than twofold. Comparison of the two tumour groups resulted in 2420 probes with expression levels differing more than twofold. In all, 344 individual genes were identified by performing a *T*-test between the two groups, followed by annotation according to the HPRD and subsequent division into eight groups depending on biological functions (Figure 2). Euclidean hierarchical clustering was performed for the 344 genes as a whole (Figure 2A), and subsequently for each of the eight subgroups (Figure 2B). The gene symbol and location in the cluster of a subset of the 344 genes is illustrated in Figure 2B. Most notable, genes encoding proteins involved in DNA replication, DNA repair, DNA binding and key regulators of the cell cycle were upregulated in Gr2, while genes involved in cell–cell adhesion, desmosome formation and apoptosis as well as several protein kinases and phosphatases, members of the Rho-Ras GTPase families and structural proteins were downregulated. Among the 344 genes identified, several were regulated by the transcription factor E2F. Therefore, we determined expression profiles on a selection of 114 genes specifically regulated by E2F (Ishida *et al*, 2001) and subsequently performed hierarchical clustering. The resulting hierarchical clustering (Figure 3Ad) was highly similar to both the hierarchical clustering of our unsupervised analysis (Figure 3Aa) and to the hierarchical clustering of genes selected by performing two independent statistical analyses between the two tumour groups (Figure 3Ab and Ac). Similarly, to investigate the possible transcriptional influence of high-risk HPV, we performed hierarchical cluster analysis on genes reported differentially expressed in several systems related to high-risk HPV infection. This included gene expression profiling of a mouse SCC model (Loercher *et al*, 2004), effect on overall gene expression associated with integration of HPV16 in cervix SCC (Alazawi *et al*, 2002), gene expression profiling of primary HPV16- and HPV18-infected early stage cervical cancer (Santin *et al*, 2005), effect of HPV18 E6 and E7 on global gene expression in an organotypic keratinocytes culture system (Garner-Hamrick *et al*, 2004), effect of HPV16 E6 and/or E7 on global gene expression in differentiating cervical keratinocytes (Nees *et al*, 2001) and, finally, effect of HPV31 on global gene expression in normal human keratinocytes (Chang and Laimins, 2000). Again, the resulting hierarchical cluster (Figure 3Ae–Aj) was practically identical compared to our unsupervised analysis (Figure 3Aa). Figure 3B illustrates the number of genes reported by others and used in this study as supporting information, and the distribution of these genes within our selection.

To test whether this division into two groups was particular to our collection of biopsies, we tested the mRNA expression of a number of genes listed in Figure 2B in a new series of anal carcinoma biopsies from six individual patients. The transcription of these genes in these biopsies also divided them into two groups with three biopsies in each group (data not shown).

Finally, we investigated expression profiling of genes selectively regulated by either CDKN2A (p16) or by E2F as reported by Vernell *et al* (2003). We found that hierarchical clustering based on CDKN2A (p16)-regulated genes could not reproduce the unsupervised clustering (Figure 3Ca), while genes regulated directly by E2F reproduced the unsupervised cluster analysis (Figure 3Cb).

**Figure 4:**
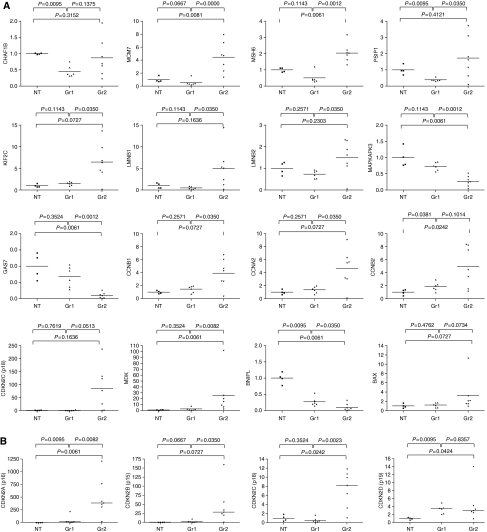
(**A**) Taqman analysis of 16 individual genes assessed relative to *β*-actin confirming the results from the microarray analysis. For all plots, the median values of each group are indicated, with all values adjusted so that the median of the NT group equals 1. Each plot is identified by the corresponding gene symbol of the target gene. *P*-values were two-sided and considered significant when <0.05. (**B**) Relative expression of the four cyclin-dependent kinase inhibitors CDKN2-A-D, assessed by microarray hybridisation. The values are adjusted so that the median of the NT groups is equal to 1.

Quantitative real-time PCR analyses confirmed the results from the microarray analysis for a selection of these genes (Figure 4).

### Immunohistochemistry and tissue microarray

**Figure 5:**
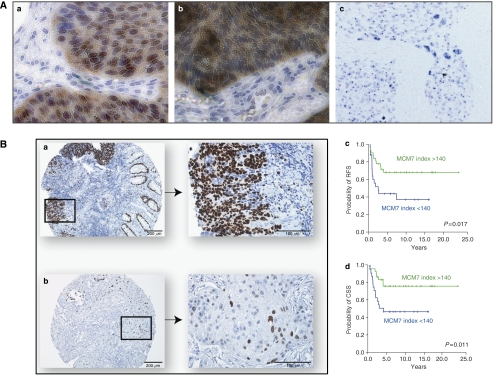
Immunohistochemical staining of MCM7 and CDKN2A proteins and ISH of HPV E7 mRNA is shown in (**A**), (**Aa**) MCM7 with predominant nuclear staining, (**Ab**) CDKN2A with nuclear and cytosolic staining and (**Ac**) ISH showing staining of predominantly nucleolar HPV E7 mRNA. (**B**) Immunohistochemical analysis of MCM7 protein expression in an anal carcinoma biopsy, illustrating one biopsy categorised as ‘high’ (upper pictures) (**Ba**) and one biopsy categorised as ‘low’ (lower pictures) (**Bb**) in the following Kaplan–Meier analysis. Kaplan–Meier estimates of RFS (upper graph) (**Bc**) and CSS (lower graph) (**Bd**) according to MCM7 protein expression among 55 patients with anal carcinomas.

Following the results from the microarray analysis, CDKN2A (p16) and MCM7 were selected as a typical gene overexpressed in Gr2 to be investigated in an independent cohort of 55 anal cancer biopsies. CDKN2A (p16) protein staining was not significantly related to survival in Kaplan–Meier analyses (log-rank test, *P*=0.70) (data not shown). However, we found that high expression of MCM7 in this cohort of anal carcinomas correlated with favourable clinical outcome (log-rank test, *P*=0.017 for probability of RFS and *P*=0.011 for probability of CSS), as illustrated in Figure 5Bb.

### *In situ* hybridisation and HPV detection

Out of 62 cases with available representative tumour tissue, E7 mRNA was detected in 34 anal carcinoma biopsies (55%). The intensity of the nucleolar staining was recorded as negative in 28 (45%), weak in 12 (19%), moderate in 8 (13%), strong in 8 (13%) and intense in 6 (10%). There was a significant association between E7 mRNA signal detected by ISH and MCM7 protein staining detected by IHC. Out of 62 anal carcinoma patients, 33.3% of those with a low MCM7 staining intensity (product <140) were HPV positive, as compared with 68.4% of those with a high MCM7 product (*P*=0.007, *χ*^2^ statistics). Also, there was a significant trend for increasing MCM7 immunostaining with increasing E7 mRNA staining intensity (recorded 0–4) (*P*=0.007 *χ*^2^ test for trend). In addition, there was a significant association between presence of HPV mRNA and high CDKN2A (p16) immunostaining (*P*=0.001).

While a high MCM7 protein staining was significantly associated with an improved CSS in 55 anal carcinoma patients treated with radiation with or without chemotherapy (Figure 5), presence of HPV mRNA assessed by ISH was not significantly related to survival in Kaplan–Meier analyses (log-rank test *P*=0.92).

## DISCUSSION

To our knowledge, this is the first description of how high-risk HPV infection influences *in vivo* global gene expression patterns in anal cancer. However, the effect of high-risk HPV on global gene expression has been studied and characterised in several model systems on different microarray platforms (Chang and Laimins, 2000; Nees *et al*, 2001; Alazawi *et al*, 2002; Vernell *et al*, 2003; Garner-Hamrick *et al*, 2004; Loercher *et al*, 2004; Santin *et al*, 2005). Several biomarkers predicting both HPV status and response to treatment for several cancers have been proposed. MCM7 and CDKN2A (p16) were proposed as biomarkers for HPV-positive head and neck cancer (Strati *et al*, 2006), while CDKN2A (p16) alone was suggested as a marker in HPV-infected oropharyngeal cancers with favourable prognosis (Weinberger *et al*, 2006). MCM7 was also proposed as an informative biomarker in cervical cancer (Brake *et al*, 2003). Both MCM7 and CDKN2A (p16) are among a number of genes regulated by the transcription factor E2F, and should therefore be transcriptionally induced in cells infected by high-risk HPV.

By investigating gene expression profiles in anal cancer biopsies, we identified two major subsets of anal cancers with unique and distinct molecular fingerprints. All cancer biopsies included in the microarray series tested positive for the presence of HPV16 mRNA by quantitative PCR. No other species of HPV was detected by PCR amplification followed by DNA sequencing in these particular biopsies. This does not reflect the distribution of HPV in Norwegian tumour samples reported by others, although HPV16 is reported to be the most common high-risk HPV in Norwegian specimens, accounting for more than 50% of all cases (Kraus *et al*, 2004; Molden *et al*, 2005). Owing to the technical limitation of the PCR/sequencing assay, we neither can exclude the presence of other subspecies of HPV within our samples, nor can we exclude the presence of viral variants, sequence disruptions or very low HPV levels that escaped detection by the methods used.

Cyclin-dependent kinase inhibitor 2A (p16) and MCM7 were significantly upregulated in one of the tumour subsets, indicating that this subset (Gr2) could be characterised by E2F-regulated transcription. This distinction could not be ascribed solely to the presence of HPV16, as there were extensive variations in both the relative amount of genomic viral DNA present and the level of viral mRNA expressed. However, the distinction could be explained by a major impact in penetrance of transcriptional regulation specifically controlled by E6 and E7, two of the genes encoded for by the HPV16 genome. Furthermore, one of the two subsets (Gr2) could be characterised by transcriptional regulation of many genes involved in biological processes similar to those controlled by E2F. Among the genes highly upregulated in Gr2, we identified several proteins involved in the initiation of DNA replication, as MCM2, MCM6, MCM7 and MCM8 (Figure 2), and three of the four cyclin-dependent kinase inhibitors, namely, CDKN2A (p16), CDKN2C (p18) and CDKN2D (p19) (Figure 4B).

To further characterise differences in the transcriptional response in the two tumour groups, we isolated genes transcriptionally controlled by CDKN2A (p16). However, cluster analysis based on these genes could not reproduce the unsupervised clustering (Figure 3C). This indicates that, although CDKN2A were highly upregulated in Gr 2, the effect of CDKN2A protein on gene expression does not follow the mRNA expression pattern of the cyclin-dependent kinase inhibitors.

Analysis of the association between MCM7 and CDKN2A (p16) protein expression, as accessed by IHC, and prognosis were performed on a separate cohort of anal carcinomas consisting of biopsies from 55 patients arranged in a tissue array. Human papillomavirus statuses of these biopsies were assessed by ISH. We found a strong correlation between high-risk HPV E7 staining and both CDKN2A (p16) and MCM7 expression, but no association between HPV expression and survival. This could, however, be due to sensitivity limitations of the HPV assay. On the other hand, elevated protein levels of MCM7 were significantly associated with favourable prognosis in patients treated with concomitant radiation and chemotherapy, both regarding RFS and CSS.

Our results suggest that transcriptional control exercised by the cyclin-dependent kinase inhibitor CDKN2A is inhibited in anal carcinomas. We also find that there is no association between CDKN2A protein expression and probability for survival. Furthermore, we find no correlation between HPV expression and survival, probably due to the limitations of the HPV assay, both regarding sensitivity and the fact that it cannot detect variants of HPV. We did, however, find a strong correlation between the MCM7 gene product (E2F regulated, HPV induced and absolute required for DNA replication) and both CSS and RFS. These findings may have clinical implications.

## Figures and Tables

**Table 1:** The histopathological diagnosis, group (based on gene expression), TNM stage and relapse status

**Biopsy no.**	**Histology**	**Group^a^**	**TNM stage, age and sex^b^**	**Relapse^c^ (months)**	**Status^d^ (months)**
ca-3	SCC	I	T_2_N_0_M_0_, 54, M	No	Alive (49)
ca-20	SCC	I	T_4_N_2_M_0_, 63, F	No	Alive (34)
ca-23	SCC	I	T_3_N_0_M_0_, 45, M	No	Alive (37)
ca-29	Paget	I	T_3_N_0_M_0_, 88, F	No	Alive (58)
ca-36	SCC, basaloid	I	T_3_N_2_M_0_, 75, F	No	Alive (25)
ca-39	SCC	I	T_2_N_0_M_0_, 78, M	No	Alive (54)
ca-11	SCC	II	T_3_N_2_M_0_, 62, F	No	Alive (50)
ca-22	SCC	II	T_2_N_0_M_0_, 56, F	No	Alive (47)
ca-24	SCC	II	T_4_N_2_M_0_, 80, F	Yes, LR (11)	Dead (12)
ca-25	SCC	II	T_2_N_0_M_0_, 48, F	No	Alive (38)
ca-34	SCC	II	T_4_N_3_M_0_, 78, F	No	Alive (37)
ca-41	SCC	II	T_3_N_0_M_0_, 81, F	Yes, DR (52)	Dead (55)
ca-47	SCC	II	T_3_N_3_M_1_, 47, M	M1 at diagnosis	Dead (6)

F=female; M=male; SCC=squamous cell carcinoma; TNM=tumour, nodes and metastases.

a Group according to unsupervised hierarchical cluster analysis.

b TNM stage according to 1987 revision. Age at diagnosis.

c LR=local relapse; DR=distant relapse; M1=distant metastases at diagnosis.

d Clinical status with time interval from diagnosis.
